# Resveratrol Prevents ROS-Induced Apoptosis in High Glucose-Treated Retinal Capillary Endothelial Cells via the Activation of AMPK/Sirt1/PGC-1*α* Pathway

**DOI:** 10.1155/2017/7584691

**Published:** 2017-10-29

**Authors:** Jun Li, Songping Yu, Jia Ying, Tianyan Shi, Peipei Wang

**Affiliations:** ^1^Department of Ophthalmology, Lishui Central Hospital, Fifth Affiliated Hospital of Wenzhou Medical University, Lishui, China; ^2^Department of Ophthalmology, Lishui Hospital Affiliated with Zhejiang University, Lishui, China; ^3^Department of Stomatology, Lishui Hospital Affiliated with Zhejiang University, Lishui, China

## Abstract

Resveratrol (RSV) is used as a protective therapy against diabetic retinopathy. However, the mechanism(s) underlying this protective effect has not been fully elucidated. Bovine retinal capillary endothelial cells (BRECs), an *in vitro* model, were used to investigate the mechanism of RSV. Our results showed that high glucose induced significant cellular apoptosis in BRECs, which was accompanied by increased intracellular levels of reactive oxygen species (ROS) and cleaved caspase-3. The glucose-induced apoptosis and ROS elevation were both inhibited by RSV. High glucose was found to decrease the levels of phosphorylated AMP-activated protein kinase (p-AMPK), which was accompanied by increased levels of Sirt1 and PGC-1*α*. These changes were reversed by RSV. We also demonstrated that AMPK regulates the modulations of Sirt1 and PGC-1*α* using specific inhibitors of AMPK and Sirt1 and small interfering RNAs of PGC-1*α*. In summary, the current study demonstrates that RSV is effective against high glucose-induced cellular apoptosis and its action is exerted via the inhibition of ROS/AMPK/Sirt1/PGC-1*α* pathway.

## 1. Introduction

Diabetic retinopathy (DR) is one of the major causes of severe vision loss and blindness in the working-age population [1]. Vision loss in DR is caused by macular edema, which is characterized by vascular leakage from increased vascular permeability secondary to the breakdown of the blood-retinal barrier (BRB) [1, 2]. The endothelium, which is a single layer of cells that covers the inner surface of all blood vessels, plays a critical role in the regulation of vascular function in the pathological processes of DR [3]. Apoptosis of endothelial cells of the retinal vasculature plays a vital role in the pathogenesis of DR [4, 5]. Thus, therapeutic strategies focus on the identification of pharmacological targets that are involved in DR-induced endothelial apoptosis.

In *in vitro* systems, high glucose (HG), an independent risk factor for diabetes, has been shown to induce apoptosis in retinal capillary endothelial cells [5, 6]. A hypothesis has been proposed that high blood glucose induces oxidative stress through the generation of excessive reactive oxygen species (ROS), which play a dominant role in the development of chronic complications caused by diabetes, including retinopathy [7, 8]. Several studies suggested that HG can lead to overproduction of ROS in endothelial cells and subsequent apoptosis [9]. Peroxisome proliferator-activated receptor-*γ* coactivator 1*α* (PGC-1*α*) is an important mediator of the metabolic effects of ROS, where PGC-1*α* activation results in the increase of mitochondrial energy metabolism and the cellular capacity to detoxify ROS, thereby reprogramming cell metabolism to maintain survival [10–13].

The AMP-activated protein kinase (AMPK) is a trimeric enzyme that contains a catalytic *α*-subunit and regulatory *β*- and *γ*-subunits [14]. AMPK acts to ameliorate the pathogenesis of metabolic disorders, including diabetes, by controlling the expression and activation of various downstream molecules [15]. In addition to regulating energy metabolism, AMPK participates in the regulation of many other cellular processes, including inflammatory responses, autophagy, and apoptosis [16, 17]. Our previous study showed that interaction between AMPK and sensor class III histone deacetylase sirtuin 1 (Sirt1), (interact to cause) caused endothelial damage and induced apoptosis in experimental diabetes [18]. Thus, AMPK/Sirt1 pathway plays an important role in microvascular damage associated with diabetes, including DR.

Resveratrol (3,5,4′-trihydroxystilbene (RSV)), a polyphenolic phytoalexin found in red wine and grape skin, has been reported to provide a wide range of health benefits such as reducing oxidative, inflammatory, and apoptotic signals [16, 19, 20]. RSV has also been found to be beneficial in the treatment of diabetes mellitus and its complications [21, 22]. The antidiabetic effects of RSV are believed to occur via the activation of AMPK and Sirt1, and subsequent reduction in the circulatory levels of proinflammatory cytokines and proapoptotic cells, along with enhancement of antioxidant defenses [23, 24]. A recent study, however, indicated that RSV improves oxidative stress and protects against diabetic nephropathy in an AMPK/Sirt1-independent manner [25]. To investigate the protective effect of RSV on DR and its dependency on AMPK/Sirt1 pathway, apoptosis was induced in bovine retinal capillary endothelial cells (BRECs) by exposure to HG and demonstrated a critical role of ROS in HG-mediated apoptosis and the regulatory role of AMPK/Sirt1/PGC-1*α* pathway in the antiapoptotic activity of RSV.

## 2. Materials and Methods

### 2.1. Reagents

Dulbecco's modified eagle's medium (DMEM), fetal bovine serum (FBS), and collagenase type II were purchased from Gibco (Los Angeles, CA, USA). Fluorescent probe 5-(and-6)-chloromethyl-2′,7′-dihydrodichlorofluorescein diacetate acetyl ester (CMH2DCFDA) was purchased from Cambridge Isotope Laboratories (Andover, MA, USA). Antibodies against cleaved caspase-3, AMPK, p-AMPK(Thr172), Sirt1, PGC-1*α*, and GAPDH were purchased from Abcam (Cambridge, UK). Horseradish peroxidase-conjugated secondary antibodies were purchased from Abcam. Double staining with Annexin V-FITC and PI kit was purchased from Roche (Shanghai, China). Resveratrol and all other reagents were purchased from Sigma-Aldrich (St. Louis, MO, USA) unless noted otherwise.

### 2.2. Cell Culture

BRECs were cultured according to previous study [26]. In brief, BRECs were cultured in low-glucose DMEM supplemented with 10% FBS, 100 U/mL penicillin and 100 mg/mL streptomycin at 37°C in a humidified atmosphere (5% CO_2_; 95% air). BRECs were identified by their expression of Von Willebrand as judged by immunocytochemical staining. The BRECs used in this project were taken from passages 4 to 6, and the cells were cultured in DMEM in the presence of D-glucose (5 or 30 mM) alone or in combination with mannitol as an osmotic control (25 mM) and H_2_O_2_ (5 mM), RSV (1, 5, 10, 20 *μ*M), N-acetylcysteine (NAC, a general ROS scavenger, 10 mM), 5-aminoimidazole-4-carboxamide ribonucleoside (AICAR, an activator of AMPK, 10 mM), 3-(2′,4′-dichlorophenyl)-7-hydroxy-4H-chromen-4-one (DCHC, a Sirt1 activator, 10 mM), compound C (an antagonist of AMPK, 10 mM), or EX527 (an inhibitor of Sirt1, 10 mM).

### 2.3. Cell Viability Assay

Cell viability was evaluated with the 3-[4.5-dimethylthiazol-2-yl]-2,5-diphenyl tetrazolium bromide (MTT) assay. BRECs (1 × 10^5^ cells) were plated in 96-well plates and cultured overnight. The cells were treated with 1, 5, 10, and 20 *μ*M RSV for 48 h. The medium was then replaced with fresh medium containing 0.5 mg/mL MTT for 4 h. After incubation, the medium was carefully removed from the plate, and dimethylsulfoxide (DMSO) was added to solubilize the formazan produced from MTT by the viable cells. The absorbance value was measured in a microplate reader (Bio-Rad Laboratories, Shanghai, China) at 490 nm. Values were expressed as a percentage relative to normal glucose (NG).

### 2.4. Measurement of Intracellular ROS Production

The level of intracellular ROS was detected by the peroxide-sensitive fluorescent probe dichlorodihydrofluorescein diacetate (DCFH-DA). DCFH-DA is converted by intracellular esterases to DCFH, which is oxidized into fluorescent dichlorofluorescein (DCF) in the presence of a proper oxidant. Samples were incubated with 2 *μ*M DCFH-DA in serum-free DMEM in the dark at 37°C with 5% CO_2_ for 30 min. After incubation, the cells were washed twice with PBS and used immediately for flow cytometry with excitation wavelength at 488 nm and emission wavelength at 525 nm. Mean fluorescence was calculated by using the program Cell Quest (Bio-Rad, USA). Each experiment was repeated three times.

### 2.5. Detection of Apoptosis by Flow Cytometry

After 24 h, cells were washed in Annexin V binding buffer and were incubated in 500 *μ*L Annexin V staining solution (1 : 50 dilution of Annexin V-phycoerythrin in Annexin V binding buffer) for 10 min in the dark at 37°C, 5% CO_2_. The cells were then washed with ice-cold PBS twice and resuspended in the binding buffer, then 10 *μ*L of propidium iodide (PI) was added for 10 min at 4°C. Flow cytometry was used to collect 5000 cells, which were sorted based on whether they were stained with PI (apoptotic or dying cells) or were unstained with either fluorochrome (live cells). All data were collected, stored, and analyzed by Multigraph software (Coulter, Miami, FL, USA).

### 2.6. RNA Extraction and Real-Time RT-PCR

Total RNA was extracted from BRECs using TRIzol reagent (Invitrogen Life Technologies, Gaithersburg, MD) and stored at −80°C. The DyNAmo Flash SYBR Green qPCR kit (Finnzymes Oy, Espoo, Finland) was used according to the manufacturer's instructions. The reaction conditions were 40 cycles of two-stage PCR consisting of denaturation at 95°C for 30 sec and annealing at 60°C for 30 sec after an initial denaturation step at 95°C for 5 sec. The primer sets are listed in Table 1).

The specificity of the amplification product was determined by performing a melting curve analysis. Standard curves were generated for each gene by using serial dilutions of known quantities of the corresponding cDNA gene template. Relative levels of target gene mRNA expression were calculated using the 2^−ΔΔCT^ method. Amplification of the target gene cDNA was normalized to *β*-actin expression.

### 2.7. Transfection of Cells with PGC-1*α* siRNA

For PGC-1*α* silencing, BRECs were transfected with 20 *μ*M of PGC-1*α* small interfering RNAs (siRNAs) by using Lipofectamine 2000 reagent (Invitrogen Life Technologies) according to the manufacturer's instructions. siRNAs were synthesized by ShineGene Molecular Biotechnology Co. Ltd. (Shanghai, China) and the sequence of siRNAs was as follows: PGC-1*α*, 5′-CCGGGGCAAATACACTCTTC-3′ and 5′-GAATTTCGGTGTGTGCGGTG-3′. BRECs were cultured in 6-well plates. Lipofectamine and siRNAs were diluted in Opti-MEM I Reduced Serum Medium (Invitrogen Life Technologies) and incubated for 10 min at room temperature. The diluted solutions were mixed and incubated for 20 min at room temperature. The mixtures were then added to each well of cultured BRECs. Six h after transfection, cells were then treated with 10 mM RSV in 30 mM glucose media for 48 h. Thereafter, cells were harvested for further experiments.

### 2.8. Protein Preparation and Western Blot

BRECs were plated at a density of 3 × 10^6^ cells/well on 6-well plates and lysed using modified RIPA buffer on ice for 30 min, and the lysates were centrifuged at 12,000 ×g for 15 min at 4°C. Proteins in the supernatant were quantified using the bicinchoninic acid (BCA) protein assay. Equal protein amounts from each sample were run in 10% sodium dodecyl sulfate polyacrylamide gel (SDS-PAGE) in a Bio-Rad miniature slab gel apparatus and transferred onto a nitrocellulose membrane. After blocking with 5% nonfat dried milk solution for 1 h, the membranes were incubated overnight at 4°C with primary antibodies for cleaved caspase 3 (1 : 500), AMPK (1 : 500), p-AMPK Thr172 (1 : 500), Sirt1 (1 : 500), and PGC-1*α* (1 : 500). Goat anti-rabbit IgG (1 : 1000) was used as the secondary antibody. To detect GAPDH expression, we used a monoclonal antibody (1 : 1000; ProteinTECH Group, Chicago, IL, USA) as an internal control to confirm equivalent total protein loading. All measures are expressed relative to the signal intensities measured in the control lanes.

### 2.9. Statistical Analysis

Data were presented in mean ± SEM. One-way analysis of variance (ANOVA) was performed followed by Tukey's post hoc test. *p* value < 0.05 was considered statistically significant. All computations were performed with the SPSS 16.0 (Chicago, IL) software.

## 3. Results

### 3.1. Cell Culture and Identification

**Figure 1:**
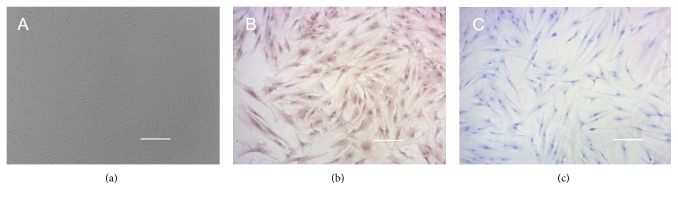
Morphology and identification of cultured BRECs. BRECs showed the typical cobblestone-shaped morphology (a) and were homogeneously positive for Von Willebrand (b) and negative for smooth muscle actin antigen (c). Scale bar indicates 25 *μ*m.

BRECs were isolated from tissues obtained from a local slaughterhouse and cultured following protocols described previously [26]. After 3-4 passages, BRECs appeared flat and assumed a cobblestone-shaped morphology (Figure 1(a)). These cells were stained positive for Von Willebrand, a molecular marker for retinal endothelial cells, with a finely granular cytoplasmic staining pattern (Figure 1(b)), and were negative for smooth muscle actin (Figure 1(c)). This immunocytochemical labeling confirms that the cultured cells are retinal capillary endothelial cells.

### 3.2. Effect of RSV on the Viability of Retinal Endothelial Cells

**Figure 2:**
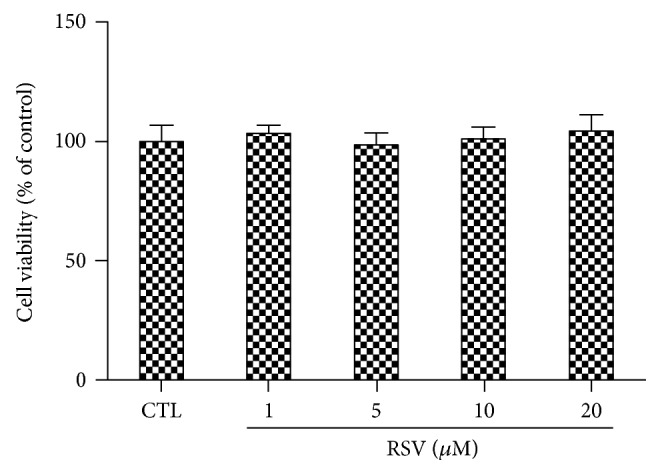
RSV has no effect on the cell viability of BRECs. After BRECs were treated with different concentrations of RSV (1, 5, 10, and 20 *μ*M) for 24 h, cell viability was measured by 3-(4,5-dimethylthiazol-2-yl)-2,5-diphenyltetrazolium bromide (MTT) assay. No significant cytotoxicity of RSV on BRECs was noted. Bars indicate the mean ± SEM of three independent experiments.

To investigate the cytotoxic effect of RSV on retinal endothelial cells, we performed an MTT assay after 24 h exposure to RSV at concentrations ranging from 1 to 20 *μ*M. Up to the highest concentration (20 *μ*M), RSV did not affect the viability of the BRECs (Figure 2). These results indicate that 20 *μ*M RSV is safe for BRECs, and thus, we used RSV at 20 *μ*M dose for subsequent studies.

### 3.3. RSV Suppresses HG-Induced BREC Apoptosis through Reduction of ROS Production

**Figure 3:**
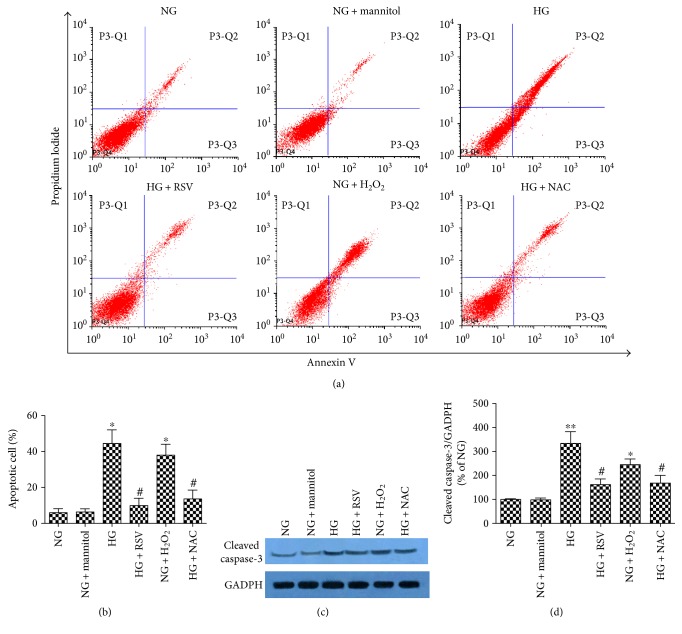
Effect of RSV on HG-induced BREC apoptosis. (a) Annexin V-FITC/PI staining of BRECs in each group. (b) Percent of cells that were PI positive. (c) Western blot analysis for cleaved caspase-3. (d) Western blot quantification. Caspase-3 levels were normalized by GADPH, and data are expressed relative to the NG mean. Bars indicate the mean ± SEM of three independent experiments. ^∗^*p* < 0.05 versus NG, ^∗∗^*p* < 0.01 versus NG, ^#^*p* < 0.05 versus HG-treated group.

**Figure 4:**
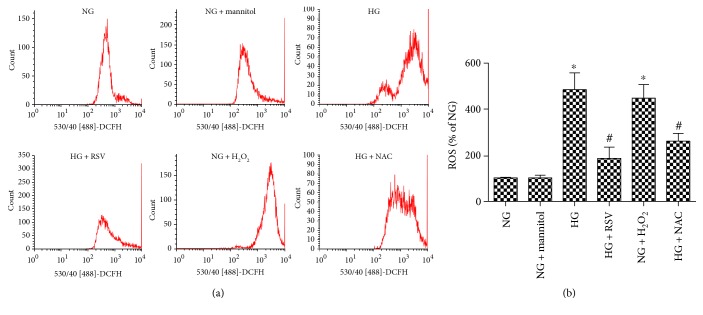
Effect of RSV on HG-induced ROS production. (a) Intracellular ROS generation in BRECs in each experimental group, identified by the fluorescent probe DCFH-DA. (b) Bars indicate the means ± SEM of three independent experiments, results are expressed as a percent of the NG mean. ^∗^*p* < 0.05versus NG, ^#^*p* < 0.05 versus HG-treated group.

BRECs were cultured with NG, NG + mannitol or HG alone, or in combination with RSV, H_2_O_2_, or NAC (Figures 3 and 4). When cultured with NG, most BRECs had low levels of Annexin V and few cells were PI positive. Corresponding rates of apoptotic cells, ROS, and cleaved caspase 3 were low. This pattern was not changed by the addition of mannitol. When cultured with HG, the number of Annexin V, PI-positive cells, levels of apoptotic cells, ROS, and cleaved caspase-3 increased. All of the HG-induced changes were normalized by addition of 20 *μ*M RSV. Consistent with the important role for the oxidative damage, incubation in NG along with H_2_O_2_ mirrors the results obtained with HG, and the ROS scavenger NAC reduced all of the changes induced by HG.

### 3.4. Effect of RSV on the AMPK/Sirt1/PGC-1*α* Pathway in High Glucose-Treated BRECs

**Figure 5:**
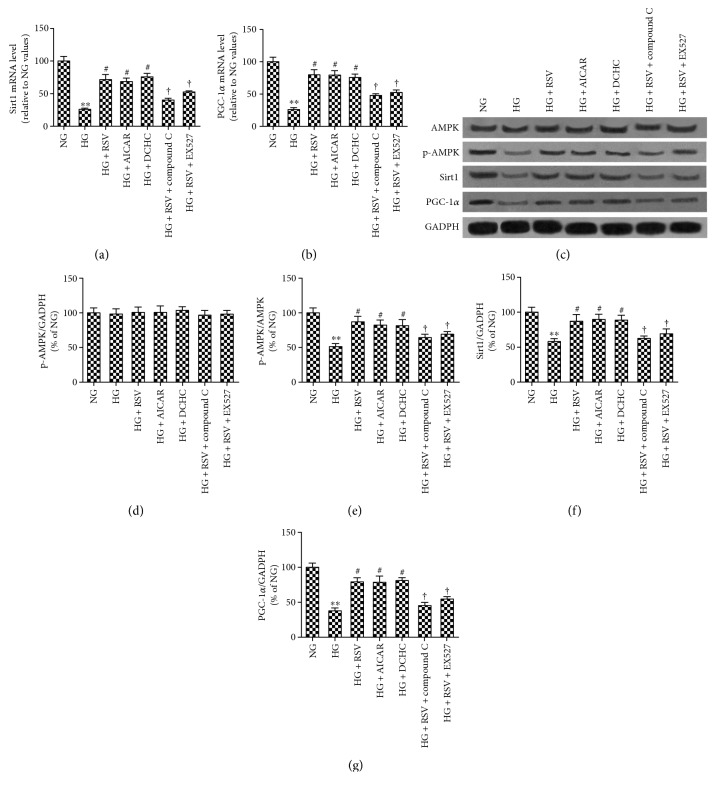
Effects of RSV on the HG-stimulated AMPK-SIRT1-PGC-1*α* axis in BRECs. (a, b) Sirt1 and PGC-1*α* mRNA expression were quantified by real-time RT-PCR in BRECs. (c) Representative Western blot analysis of AMPK, phospho-AMPK Thr^172^, Sirt1, PGC-1*α*, and GADPH levels. (d–g) Bars indicate the mean ± SEM from three independent experiments. GADPH was set as a control for normalization (*n* = 3). ^∗∗^*p* < 0.01 versus NG, ^#^*p* < 0.05 versus HG-treated group, ^†^*p* < 0.05 versus HG plus RSV-treated group.

Previous studies have shown that the AMPK/Sirt1/PGC-1*α* pathway plays an important role in the induction of ROS-induced apoptosis in diabetes [18, 27, 28]. mRNA by the 2^−ΔΔCT^ method and protein levels by Western blot were therefore quantified. As shown in Figure 5, mRNA levels of Sirt1 and PGC-1*α* were substantially reduced in BRECs treated with HG as compared to NG (*p* < 0.05 for each). By Western blot analysis, protein levels p-AMPK, Sirt1, and PGC-1*α* were substantially reduced in BRECs treated with HG as compared to NG (all *p* < 0.05). In comparison, nonphosphorylated AMPK levels were unchanged (*p* > 0.05). When HG-cultured BRECs were treated with RSV, mRNA levels of Sirt1 and PGC-1*α* approached the NG level and the protein levels of p-AMPK, Sirt1, and PGC-1*α* protein were also increased to levels near BREC cells cultured in NG.

To explore the relationship between the antiapoptotic effect of RSV and the AMPK/Sirt1/PGC-1*α* pathway, additional experiments using the AMPK activator AICAR and the Sirt1 activator DCHC were performed. Similar to RSV, addition of AICAR or DCHC to BRECs grown in HG resulted in increased levels of p-AMPK, Sirt1, and PGC-1*α* protein. In comparison, addition of compound C (an antagonist of AMPK) and EX527 (an inhibitor of Sirt1) reversed the effect of RSV.

### 3.5. RSV Mitigates ROS-Induced BREC Apoptosis by Modulating AMPK/Sirt1/PGC-1*α* Pathway

**Figure 6:**
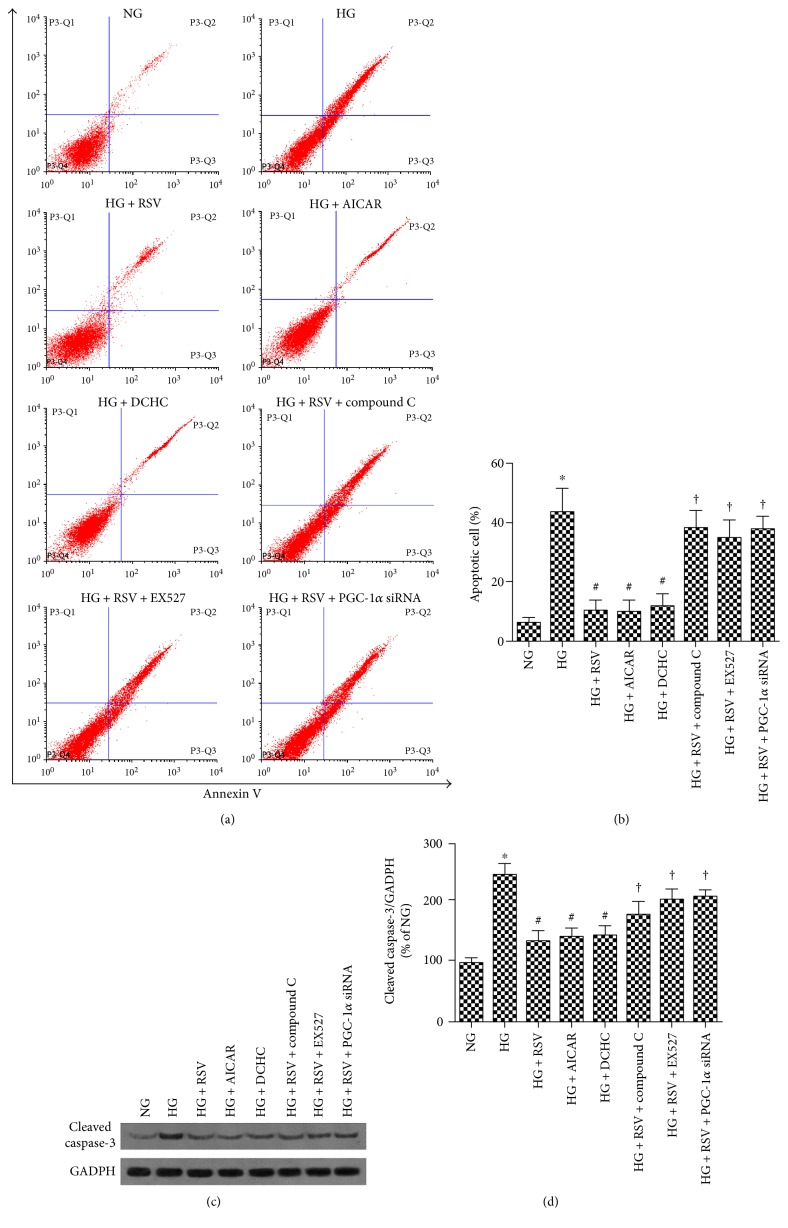
The role of AMPK-SIRT1-PGC-1*α* in RSV treatment of HG-induced BREC apoptosis. (a) Annexin V-FITC/PI staining of BRECs in each experimental group. (b) Percent of cells that were PI positive. (c) Western blot analysis for cleaved caspase-3. (d) Western blot quantification. Caspase-3 levels were normalized by GADPH, and data are expressed relative to the NG mean. Bars indicate the mean ± SEM of three independent experiments. ^∗^*p* < 0.05 versus NG, ^#^*p* < 0.05 versus HG-treated group, ^†^*p* < 0.05 versus HG plus RSV-treated group.

**Figure 7:**
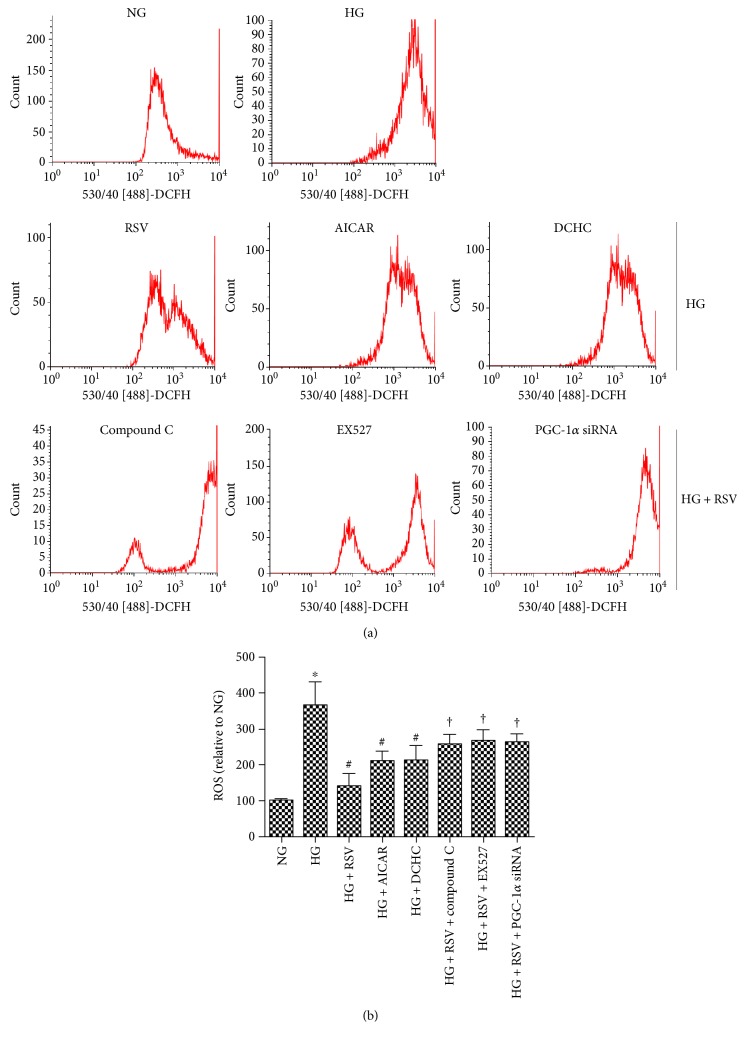
The role of AMPK-Sirt1-PGC-1*α* in RSV treatment of HG-induced ROS production. (a) Intracellular ROS generation in BRECs in each experimental group, identified by the fluorescent probe DCFH-DA. (b) Bars indicate the means ± SEM of three independent experiments; results are expressed as a percent of the NG mean. ^∗^*p* < 0.05 versus NG, ^#^*p* < 0.05 versus HG-treated group, ^†^*p* < 0.05 versus HG plus RSV-treated group.

To further explore RSV inhibition of ROS-induced BREC apoptosis through the AMPK/Sirt1/PGC-1*α* pathway, siRNA was used to reduce PGC-1*α* levels and specific inhibitors of AMPK and Sirt1. As shown in Figures 6 and 7, culture of cells in HG as compared to NG increased the levels of Annexin V and the number of PI-positive apoptotic cells. HG also increased levels of ROS and cleaved caspase-3 compared with NG. These increases were reversed when 20 *μ*M RSV was administered to HG-cultured cells. Inclusion of the AMPK agonist AICAR or the Sirt1 agonist DCHC mirrored the beneficial effect of 20 *μ*M RSV on HG-induced ROS production, cell apoptosis, and cleaved caspase-3 protein expression. In comparison, the beneficial effects of RSV were abolished by inhibitors of AMPK or Sirt1 or by treatment with PGC-1*α* siRNA. These results are consistent with a model in which RSV mitigates ROS-induced BREC apoptosis by modulation of the AMPK/Sirt1/PGC-1*α* pathway.

## 4. Discussion

In this study, we cultured BRECs in HG medium to mirror some features of DR related to endothelial cell damage. HG increased ROS levels and induced BREC apoptosis. These changes were reversed by RSV, a derivative of grape skin that can reduce oxidative damage, inflammation, and cellular apoptosis [29]. In BRECs, RSV reduced ROS levels, cleaved caspase-3, and apoptosis. The current study demonstrated that the beneficial effects of RSV could be mirrored by activators of the AMPK/Sirt1/PGC-1*α* pathway and could be blocked by agents that interfere with AMPK/Sirt1/PGC-1*α* signaling.

Microvascular changes, including thickening of capillary basement membranes, apoptosis of microvascular cells, loss of pericytes, and acellular capillary formation, are major features of DR [2]. Vascular lesions resulting from BRB breakdown are primary causes of vision loss in DR [1, 2]. Both human and animal studies indicated that microvascular apoptosis plays a crucial role in the development of early lesions [3, 30]. Although retinal microvascular cell loss plays a critical role in the pathogenesis of DR [3, 5], the mechanisms by which HG induces endothelial cell apoptosis in DR are not fully understood. In the present study, BREC culture system was used to replicate the impact of HG on endothelial cells and to verify prior findings [4, 31] that HG induces ROS production and apoptosis.

Brownlee proposed that mitochondrial production of ROS in response to hyperglycemia may be a key initiating step in the pathogenesis of diabetes, including DR [8]. Other studies have established a close relation between intracellular ROS production and retinal endothelial cell apoptosis [9, 32, 33]. In our BREC system, we confirmed that HG stimulates ROS production leading to cleaved caspase-3 expression and apoptosis. These changes induced by HG were blocked by the antioxidant NAC, but were mirrored in BRECs cultured in NG along with H_2_O_2_. These supported the use of antioxidants as a treatment for DR to target intracellular ROS production. RSV is a potent antioxidant [34, 35] that evidence was found to support the effects of RSV in diabetes and other ocular diseases [23, 29, 36, 37]. Tennen et al. proposed that RSV acts by activation of AMPK and Sirt1 [16, 38, 39], which is consistent with our results. AMPK is a serine/threonine protein kinase, which may sense regulated metabolic homeostasis by energy deficiency through an increased AMP/ATP ratio [15] through controlling the transcriptional regulation of certain genes such as PGC-1*α* and NF-*κ*B [38, 40, 41]. Cantó and Auwerx reported that AMPK regulates transcription through direct events (i.e., phosphorylation of transcriptional regulators) and also indirectly by increasing NAD+ and inducing Sirt1 activity [42]. Our results are in line with the model in which RSV acts through the AMPK/Sirt1/PGC-1*α* pathway to modulate the negative effects of HG on BRECs and support future efforts towards the use of RSV to ameliorate the cellular damage induced by diabetes.

## 5. Conclusion

Our data demonstrate that RSV acts to reduce intracellular ROS through the activation of AMPK/Sirt1/PGC-1*α* pathway and thereby suppresses apoptosis in HG-treated retinal capillary endothelial cells. This study provides insight into the application of RSV as an effective treatment for the early stages of progression in DR.

## Figures and Tables

**Table 1:** Primer details.

Prime	Forward (5′-3′)	Reverse (5′-3′)	Amplicon size (bp)
Sirt1	CCCTGAAAGTAAGACCAGTAGCAC	ACAGCAAAGTTTGGCATATTCAC	182
PGC-1*α*	CACTCTTCCACAGATTCCGACC	GACTGGGATGACCGAAGTGC	142
*β*-Actin	TCCTGCGTCTGGACCTGG	TGATGTCACGGACGATTTCC	114
